# Therapeutic effects of *Crataegus monogyna* inhibitors against breast cancer

**DOI:** 10.3389/fphar.2023.1187079

**Published:** 2023-04-27

**Authors:** Girish Meravanige Basavarajappa, Abdur Rehman, Predeepkumar Narayanappa Shiroorkar, Nagaraja Sreeharsha, Md. Khalid Anwer, Bandar Aloufi

**Affiliations:** ^1^ Department of Biomedical Sciences, College of Medicine, King Faisal University, Al-Hofuf, Saudi Arabia; ^2^ College of Life Sciences, Northwest A&F University, Yangling, China; ^3^ Department of Pharmaceutical Sciences, College of Clinical Pharmacy, King Faisal University, Al-Hofuf, Saudi Arabia; ^4^ Department of Pharmaceutics, Vidya Siri College of Pharmacy, Bangalore, India; ^5^ Department of Pharmaceutics, College of Pharmacy, Prince Sattam Bin Abdulaziz University, Al-Alkharj, Saudi Arabia; ^6^ Department of Biology, College of Science, University of Hail, Hail, Saudi Arabia

**Keywords:** network pharmacology, active compounds, *Crataegus monogyna*, breast cancer, microarray data, molecular docking

## Abstract

Breast cancer is a silent killer disorder among women and a serious economic burden in healthcare management. Every 19 s, a woman is diagnosed with breast cancer, and every 74 s, a woman worldwide passes away from the disease. Despite the increase in progressive research, advanced treatment approaches, and preventive measures, breast cancer rates continue to increase. This study provides a combination of data mining, network pharmacology, and docking analysis that surely could revolutionize cancer treatment by exploiting prestigious phytochemicals. *Crataegus monogyna* is a small, rounded deciduous tree with glossy, deeply lobed leaves and flat sprays of cream flowers, followed by dark red berries in autumn. Various studies demonstrated that *C. monogyna* is therapeutically effective against breast cancer. However, the particular molecular mechanism is still unknown. This study is credited for locating bioactive substances, metabolic pathways, and target genes for breast cancer treatment. According to the current investigation, which examined compound–target genes–pathway networks, it was found that the bioactive compounds of *C. monogyna* may operate as a viable solution against breast cancer by altering the target genes implicated in the disease pathogenesis. The expression level of target genes was analyzed using GSE36295 microarray data. Docking analysis and molecular dynamic simulation studies further strengthened the current findings by validating the effective activity of the bioactive compounds against putative target genes. In summary, we propose that six key compounds, luteolin, apigenin, quercetin, kaempferol, ursolic acid, and oleanolic acid, contributed to the development of breast cancer by affecting the MMP9 and PPARG proteins. Integration of network pharmacology and bioinformatics revealed *C. monogyna’s* multitarget pharmacological mechanisms against breast cancer. This study provides convincing evidence that *C. monogyna* might partially alleviate breast cancer and ultimately lays a foundation for further experimental research on the anti-breast cancer activity of *C. monogyna*.

## 1 Introduction

Breast cancer is a frequently diagnosed cancer and an ongoing challenge that is liable for the death of 685,000 women globally (Ganesan et al., 2022; Kaushik et al., 2022). Despite countless initiatives, breast cancer remains a severe burden on society (Noor et al., 2021). According to recent studies, increased body weight, lack of exercise, and alcohol intake are major risk factors for breast cancer (Hirose et al., 1995; Feigelson et al., 2004). Additionally, it has been noted that the condition is now being diagnosed in younger women. Breast cancer in males is a relatively rare disease with an incidence rate of < 1% of all breast cancer occurs in males (Leon-Ferre et al., 2018; Gucalp et al., 2019). The higher mortality risk in females is due to a significantly greater proportion of stromal and epithelial tissues and less fatty adipose tissue (Nazari and Mukherjee, 2018; Hieken et al., 2022). Breast cancer comprises a heterogeneous group of neoplasms with distinct probabilities of relapse, molecular phenotypes, morphologies, responses to therapy, and overall survival (Kalinowski, 2019; Rakha and Pareja, 2021). The development of targeted therapy has been the primary focus of modern cancer research. Using plants and their phytochemicals to promote well-being is as old as humanity. The tremendous richness of natural chemicals observed in plants offers exceptional possibilities for developing novel medications. Nowadays, scientists utilize multiple approaches to find the biological role of these prestigious candidates to serve as novel leads in cancer treatment. These old weapons are a novel fighter in the new war against breast cancer.

*Crataegus monogyna*, commonly known as hawthorn, is a round appearing medicinal tree with a cream flower that turns dark red in autumn (Fichtner and Wissemann, 2021; Martinelli et al., 2021; Belabdelli et al., 2022). *C. monogyna* is a fantastic source of active ingredients, including a wide range of polyphenols, including epicatechin, procyanidins, isoquercitrin, hyperoside, and chlorogenic acid, as well as a wide range of triterpene acids, including ursolic acid and oleanolic acid, and many others (Bahorun et al., 1994; Nabavi et al., 2015). The anti-oxidative and cytotoxic effects of hawthorn’s bioactive component on the human laryngeal cancer cell line were examined in a human laryngeal carcinoma cell line (Belščak-Cvitanović et al., 2014). The outcomes showed a significant reduction in radical species and cell viability associated with various dosages and times of administration.

Network pharmacology is a new frontier in systematic drug research that can elucidate the drug actions and their interaction with multiple targets (Noor et al., 2022a; Rehman et al., 2022a; Qasim et al., 2023). This approach is considered a new trend toward combining computational, experimental, and clinical approaches to increase the clinical efficacy of drugs (Hopkins, 2008). This multitarget approach is a tool for solving complex molecular puzzles that have long perplexed researchers. Due to its ability to provide a comprehensive understanding of systems biology through network theories, it has been referred to as the “next paradigm in drug development.” Recently, Batool et al. (2022) employed bioinformatics and network pharmacology to elucidate the anticancer effect of *Fumaria indica* for treating liver cancer. A wealth of studies have reported the application of network pharmacology in different domains, including cardiovascular diseases, neurodegenerative diseases, cancer, and many others. Similarly, Noor et al. (2022b) combined network pharmacology with docking studies to uncover the multicomponent effect of *Abrus precatorius* L. as a novel therapeutic option for type II diabetes.

To our knowledge, this is the first study to integrate bioinformatics with network pharmacology to analyze the multitarget pharmacological mechanisms and reveal the potential targets and active ingredients of *C. monogyna* for breast cancer. This approach constructs multicomponent and multitarget models to provide a better understanding of the intricate interactions between active compounds and target proteins from a network perspective. Additional methods, including microarray data, survival analysis, and molecular docking studies, were used to validate the findings. The results were further complemented by all-atom molecular dynamic (MD) simulation for 100 ns to investigate the stability, conformational changes, and interaction mechanism of target proteins when complexed with the proposed compounds. These findings highlight the predicted therapeutic targets may be potential targets of *C. monogyna* for the treatment of breast cancer. Further *in vivo* and *in vitro* studies are mandatory to unveil the pharmacokinetics and biosafety profile to track the candidature status of *C. monogyna* in breast cancer.

## 2 Materials and methods

### 2.1 Screening of active compounds

The identification of active compounds is a preliminary step in network pharmacology to examine the anticancer effect of *Crataegus monogyna*. A detailed literature search was carried out using Google Scholar, Google, and PubMed. Later, different public repositories were searched using keyword “*Crataegus monogyna*” for retrieval of *C. monogyna*-related active compounds. The Traditional Chinese Medicine System Pharmacology (TCMSP) (Ru et al., 2014), KNApSAcK (Nakamura et al., 2013), and Indian Medicinal Plants, Phytochemicals, and Therapeutics (IMPPAT) (Mohanraj, 2018) databases were used in the current study to obtain compound-related data. The compounds obtained from literature searches and databases were then submitted to the Swiss ADME to predict their oral bioavailability (OB). The term OB refers to the fraction of an orally administered drug that enters the systemic circulation. In contrast, a drug that is given intravenously is readily available in the bloodstream and can be quickly distributed via systemic circulation to its intended pharmacological site. In the context of drug design and development, drug-likeness (DL), including as transporter effects, metabolic stability, permeability, and solubility, plays a crucial role. These properties can influence various aspects of drug behavior, such as its metabolism, oral bioavailability, and potential toxicity. Following this, the DL of active compounds was predicted using MolSoft. Later, a filter was applied to pharmacokinetics data and only those active compounds that which met the criteria of OB ≥ 30% and DL ≥ 0.18 (Noor et al., 2022b) were selected for subsequent analysis.

### 2.2 ADMET profiling

Evaluating absorption, distribution, metabolism, excretion, and toxicity (ADMET) characteristics is crucial when assessing the pharmacological effects of the active compounds. In the past, many drug candidates could not meet the requirements for clinical trials; therefore, *in silico* prediction is crucial in the preliminary prediction. The ADME profile directly influences the blood–brain barrier, gastrointestinal environment, hydrophobicity, and physiochemical properties of a substance before it is eliminated from the body in feces and urine. admetSAR 2.0 (Cheng et al., 2012) and Swiss ADME (Daina et al., 2017) were employed for ADME analysis. Computational tools have made it possible to access the safety profile of the required ligand molecules and quantify their toxicity. Carcinogenicity, immunotoxicity, mutagenicity, and cytotoxicity were then quantified using a toxicity profile. The Protox-II tool (Banerjee et al., 2018) was employed to analyze the toxicity of ligand molecules.

### 2.3 Target identification of druggable ingredients and breast cancer

After identifying active compounds, the selected compounds were subjected to the STITCH database (Kuhn et al., 2007) and SwissTargetPrediction (Gfeller et al., 2014). STITCH databases yielded a list of target genes against each compound. Only those target genes were used for further analysis, having a combined score ≥ 0.4. SwissTargetPrediction is a simple but useful tool for predicting the most probable protein targets of active compounds. Similarly, the same search criteria were used in Swiss target prediction, and those target genes were selected with a probability ≥ 0.4 (Noor et al., 2022b). The target proteins obtained from the SwissTargetPrediction and STITCH database were merged into a single file for further analysis.

The disease-related proteins were retrieved from Online Mendelian Inheritance in Man (OMIM) (Hamosh et al., 2000) and GeneCard (Safran, 2010). Both databases provide a constantly updated list of human genes, allowing for correlating these genes with diseases and developing new diagnostic and treatment approaches. The duplicated genes were disregarded, and UniProtKB was used to determine the common names of the target proteins (Boutet et al., 2007). Lastly, a Venn diagram was plotted to identify the genes that overlapped between the active compounds and breast cancer. These overlapped genes were considered as key targets.

### 2.4 GO enrichment and KEGG pathway analysis

The overlapped targets were submitted to the DAVID database (Dennis et al., 2003) for KEGG and gene ontology (GO) pathway analysis. GO describes gene products in three different domains: molecular function (MF), biological processes (BP), and cellular components. Pathway analysis lies at the heart of systems biology. Pathway enrichment analysis helps researchers gain mechanistic insight into key targets generated from network pharmacology. A *p*-value < 0.05 filter was applied to the GO terms and KEGG pathways to obtain statistically significant results. Later, the ggplot2 package of R was employed to visualize the top 20 significant GO terms and top 20 significant KEGG pathways.

### 2.5 Network construction

In the context of network pharmacology, the relationship between target proteins and active compounds is established based on the ability of a given compound to target multiple proteins. A compound–target interaction network is constructed to investigate the molecular mechanisms underlying the therapeutic effects of active ingredients while minimizing potential side effects. This network enables a deeper analysis of the action mechanisms of the active compounds at the molecular scale. Comprehensive filtering is a crucial aspect of network pharmacology to fully understand the multicomponent effects of such a network. This approach can maximize the therapeutic effects while minimizing potential adverse effects associated with multitarget drug design. Unfortunately, owing to the overwhelming number of interactions, it is challenging to experimentally analyze all potential interactions between target proteins and their associated active ingredients. In light of this, computational modeling—particularly network approaches—offers a compelling alternative. Cytoscape (version 3.9.0, Boston, MA, United States) (Shannon et al., 2003) was used to predict the interaction between *C. monogyna*-related compounds and target proteins. In a compound–target network, the edges indicate relationships between compounds and targets, while the nodes represent compounds and their corresponding targets. Later, a compound–target–pathway was constructed using Cytoscape (version 3.9.0, Boston, MA, United States) (Shannon et al., 2003) to investigate the multitarget effect of *C. monogyna*-related compounds on breast cancer.

### 2.6 Identification of hub genes

The most fundamental molecular processes that underlie cellular life depend on protein–protein interactions (PPIs), which are frequently disturbed in disease states. The overlapped targets were subjected to the Search Tool for the Retrieval of Interacting Genes/Proteins (STRING) (Von Mering et al., 2005) for PPI network construction. STRING is a biological database and online resource for PPIs that have been observed and predicted in the past. Information from various sources, including experimental data, computational prediction techniques, and open text collections, is included in the STRING database. The PPI was then imported to Cytoscape (version 3.9.0, Boston, MA, United States) (Shannon et al., 2003) to identify hub genes. Hub genes have a high degree of connectivity in a network. The cytohubba plugin in Cytoscape was used to obtain proteins that are highly correlated with each other. The top 10 genes based on the degree method were selected and considered for further analysis.

### 2.7 Validation using gene expression data and molecular docking

To validate the current study’s findings, the gene expression data were obtained from the Gene Expression Omnibus (GEO) database (Clough and Barrett, 2016). GEO is a public repository of high-throughput gene expression data, microarrays, chips, and hybridization arrays. In the current study, the GSE36295 data set was downloaded from the GEO database. The GSE36295 data set contains microarray data obtained from breast cancer and healthy individuals. The GSE36295 data set comprises 50 samples (5 healthy individuals and 45 affected individuals). Later, the limma package of R was used to screen differentially expressed genes (DEGs). Limma, which includes effective tools for reading, normalizing, and interpreting gene expression data, has evolved to be the best option for acquiring DEGs through differential expression analysis of high-throughput data and microarray. Screening conditions were set as adj. *p-*value < 0.05 and |log(FC)| ≥ 1, |log(FC)| < −1.0. The ggplot2 package of R was used to construct a volcano plot to visualize significant and non-significant genes. Later, DEGs were compared with hub genes, and common genes were selected for further analysis.

Molecular docking was then used to predict the binding affinity and mode of action of a small molecule ligand with a target protein (Muhammad et al., 2021; Hassan et al., 2022a; Khan et al., 2022). The common genes were then docked with target proteins to predict binding affinity among active compounds and target proteins. The target protein structure was retrieved from the Protein Data Bank (PDB). PDB is a single worldwide archive of the 3D structures of nucleic acids and proteins. It contains protein data obtained by nuclear magnetic resonance (NMR) spectrometry and X-ray crystallography submitted by researchers from all over the world. After the refinement of protein structures, the binding pockets were explored through the CASTp tool (Tian et al., 2018). Later, PyRx (Dallakyan and Olson, 2015) was used for protein–compound docking. A list of docked complexes was generated using docking, but only those complexes with the highest absolute value of root mean square deviation (RMSD) and binding energy (kcal/mol) were considered. Lastly, Discovery Studio (Studio, 2008) and ChimeraX (Goddard et al., 2018) were used to explore interactions between compounds and target proteins.

### 2.8 Molecular dynamics (MD) simulation

Molecular dynamics (MD) simulations are an important methodology for examining the stability and compactness of docked complexes (Aslam et al., 2021; Rehman et al., 2022b; Noor et al., 2022c). The Desmond v3.6 program was used in the current study to validate the finding of docking studies (Mohankumar et al., 2020). The MD simulation was run at 100 ns under thermodynamical conditions, including temperature, pressure, density, and applied volume. Using ensembles, the entire system was annealed and reached equilibrium. The structural changes in docked complexes were investigated in the final production stages. To assess the degree of structural changes, the trajectories of docked complexes were exposed to several generalized parameters, including root mean square fluctuation (RMSF), root mean square deviation (RMSD), polar surface area (PSA), and radius of gyration (Rg).

## 3 Results

### 3.1 Screening of active compounds and key targets

The data on *C. monogyna* -related compounds were not available in the TCMSP database. Therefore, the KNApSAcK and the IMPPAT databases were searched for retrieval of plant-related compounds. Virtual screening of *C. monogyna*-related compounds yielded six compounds: apigenin, luteolin, quercetin, kaempferol, ursolic acid, and oleanolic acid (Table 1). These six compounds fulfill the OB ≥ 30% and DL ≥ 0.18 criteria. The ADMET analysis of bioactive compounds reveals no adverse effect of screened compounds (Table 2). P-glycoprotein substrates, Caco-2 permeability, and BBB penetration yielded favorable results, indicating the ability of bioactive compounds to be used as a novel therapeutic agent. All of the predicted compounds were found to be non-toxic, even when tested for multiple types of toxicity, and no compound displayed toxic behavior.

**TABLE 1:** Physiochemical properties, 2D structure, and PubChem IDs of final compounds.

Molecule name	Molecular	Oral bioavailability (OB)	Drug likeness (DL)	Structure	PubChem IDs
	weight (MW)				
Apigenin	270.24	0.55	0.39	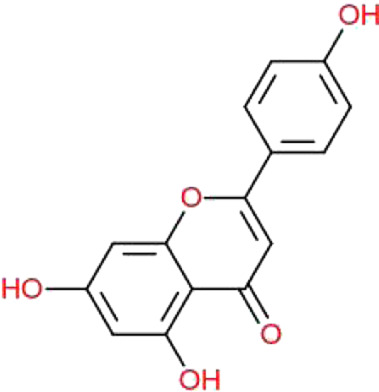	5280443
Luteolin	286.24	0.55	0.38	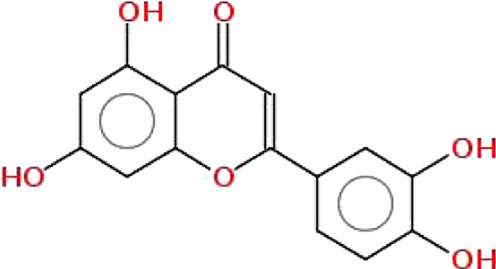	5280445
Quercetin	302.23	0.55	0.52	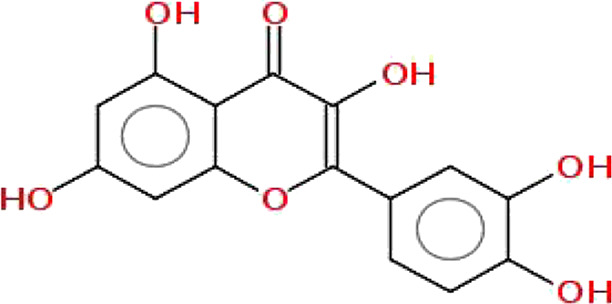	5280343
Kaempferol	286.24	0.55	0.50	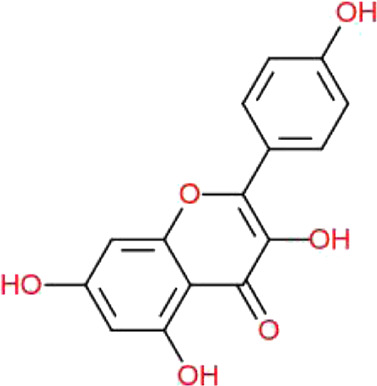	5280863
Ursolic acid	456.7	0.85	0.66	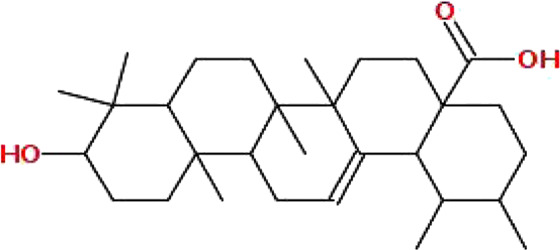	64945
Oleanolic acid	456.7	0.85	0.37	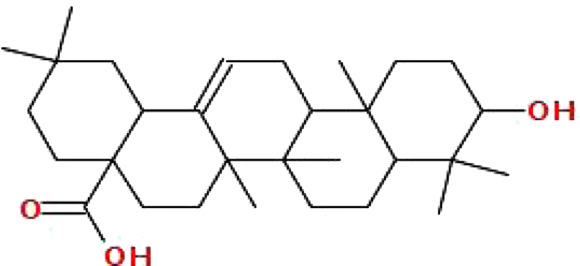	10494

**TABLE 2:** ADMET profiling of selected compounds.

Compound	Luteolin	Apigenin	Quercetin	Kaempferol	Ursolic acid	Oleanolic acid
Blood–brain Barrier	✗	✗	✗	✗	✗	✗
Caco-2 permeability	High	Low	Low	Low	High	Low
Pgp-inhibitor	✗	✗	✗	✗	✗	✗
Pgp-substrate	✗	✗	✗	✗	✗	✗
CYP1A2 inhibitor	✗	✓	✗	✓	✗	✗
CYP1A2 substrate	✗	✗	✗	✗	✗	✓
CYP2C19 inhibitor	✓	✗	✗	✗	✓	✗
CYP2C19 substrate	✗	✗	✓	✗	✗	✗
CYP2C9 inhibitor	✗	✗	✗	✗	✗	✗
CYP2C9 substrate	✗	✗	✓	✗	✗	✗
CYP3A4 inhibitor	✓	✓	✗	✓	✗	✗
CYP3A4 substrate	✗	✗	✗	✗	✓	✗
Acute toxicity	Non-toxic	Non-toxic	Non-toxic	Non-toxic	Non-toxic	Non-toxic
AMES toxicity	Non-toxic	Non-toxic	Non-toxic	Non-toxic	Non-toxic	Non-toxic

### 3.2 Network analysis

**FIGURE 1:**
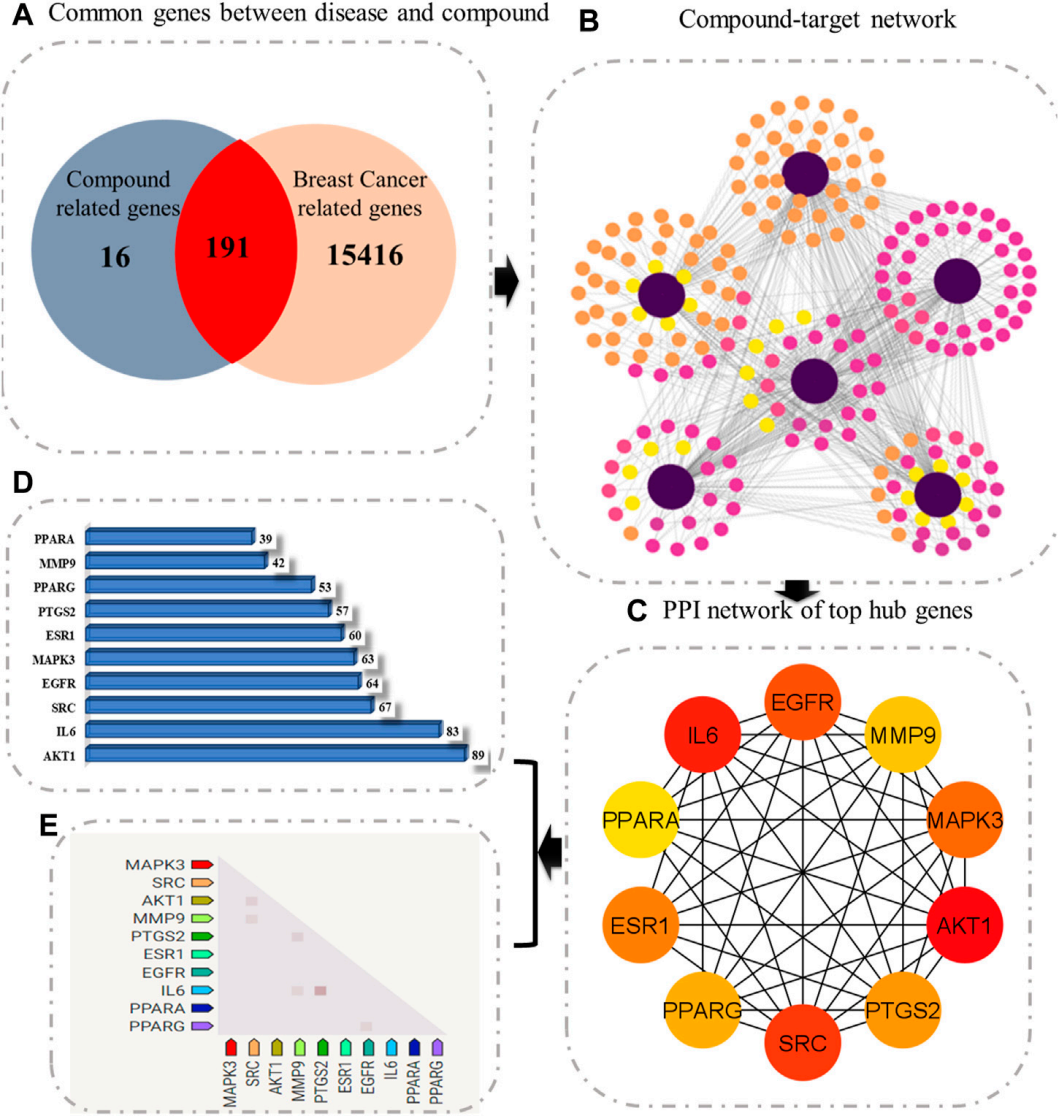
Schematic diagram representing the overall methodology for deciphering the multitarget pharmacological mechanism of *C. monogyna*. **(A)** Venn diagram representing the common genes between plant-related compounds and disease. **(B)** Compound–target network. The size and color of the nodes represent their degree of connectivity. The higher the degree, the greater the size of the node. **(C)** Ranking of key genes based on degree of connectivity. **(D)** Bar plot representing the degree of hub genes. **(E)** Observed expression hub genes in humans.

Later, the compound–target network was constructed by merging active compounds to the target genes. The main purpose of constructing a compound–target network was to comprehend the multitarget effect of *C. monogyna*-related compounds on breast cancer (Figure 1). The compound–target network consists of 600 nodes and 217 edges. The nodes in the compound–target network represent compounds and target proteins, and the degree of connectivity reflected the significance of target proteins and their associated active compounds in the network. The size and color of nodes become large and darker with increased degree of connectivity. The solid lines in the network signify the interconnection between nodes. Furthermore, overlapped genes between compound-related and disease-related targets were identified using a Venn diagram. A total of 191 genes were found to be common in both compound- and disease-related targets. These compounds were then considered key targets and selected for subsequent analysis.

The PPI network of key targets was constructed using the STRING database. The PPI network was then imported to Cytoscape for identification of hub genes. The top 10 genes based on degree of connectivity were selected and considered as the hub genes. The degree of connectivity is the number of connections or edges the node has to other nodes. These hub genes have the highest degree of connectivity in the PPI network: AKT1(89), IL6(83), SRC(67), EGFR(64), MAPK3(63), ESR1(60), PTGS2(57), PPARG(53), MMP9(42), and PPARA (39) (Figure 1C). The MNC, MCC, betweenness, closeness, and clustering coefficient of predicted hub genes are presented in Table 3.

**TABLE 3:** Degree, MNC, MCC, betweenness, closeness, and clustering coefficient of top 10 hub genes.

Gene name	Degree	MNC	MCC	Betweenness	Closeness	Clustering coefficient
*AKT1*	89	89	2.17E+08	5025.418	137.0833	1
*IL6*	83	83	2.16E+08	4385.023	134	1
*SRC*	67	67	2.16E+08	2447.815	124.75	1
*EGFR*	64	64	2.10E+08	2409.659	124.0833	1
*MAPK3*	63	63	2.09E+08	2071.007	122.75	1
*ESR1*	60	60	2.05E+08	2747.78	121.3333	1
*PTGS2*	57	57	1.99E+08	1767.963	120.25	1
*PPARG*	53	52	1.61E+08	1602.4	117.5833	0.944444
*MMP9*	42	42	1.49E+08	993.0264	111.75	0.866667
*PPARA*	39	39	1.06E+08	1107.025	109.5833	0.866667

### 3.3 GO enrichment and KEGG pathway analysis

**FIGURE 2:**
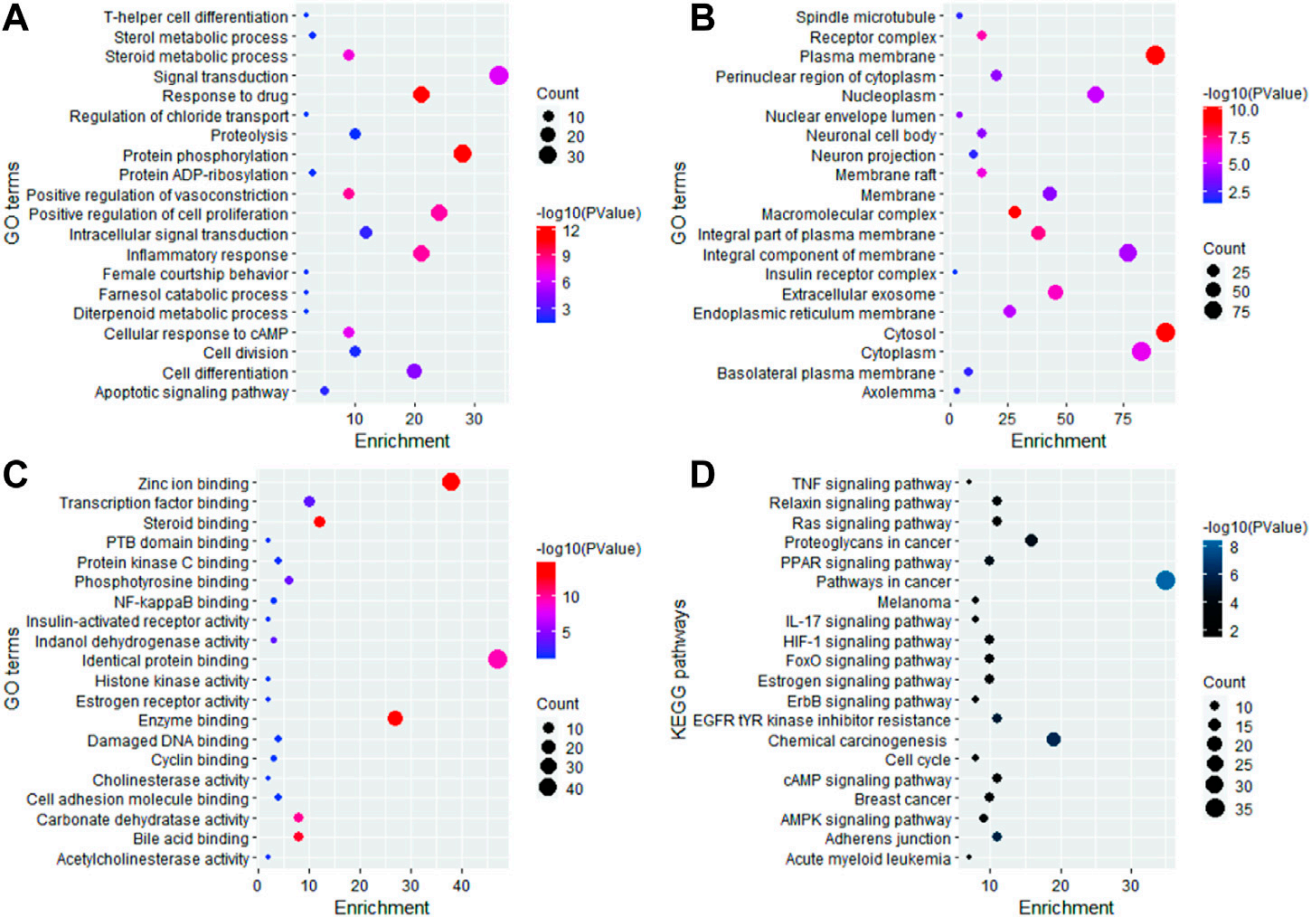
Graphical representation of the top 20 significant GO terms and KEGG pathways.

The 191 key targets were submitted to DAVID for function enrichment analysis and pathway analysis. The GO terms were mainly categorized into BP, CC, and MF. GO and KEGG pathway analysis revealed that target proteins were mainly involved in 97 BP terms, 52 CC terms, 110 MF terms, and 74 KEGG pathways. The GO enrichment analysis in terms of BP revealed that key targets were mainly enriched in signal transduction, protein phosphorylation, positive regulation of cell proliferation, response to drug, inflammatory response, cell differentiation, cell division, proteolysis, positive regulation of vasoconstriction, steroid metabolic process, cellular response to cAMP, and apoptotic signaling pathway (Figure 2A). In case of CC, the key targets were mainly concentrated in plasma membrane, cytosol, macromolecular complex, integral part of plasma membrane, receptor complex, extracellular exosome, membrane raft, cytoplasm, nucleoplasm, and endoplasmic reticulum membrane (Figure 2B). In terms of MF, the overlapped genes were mainly involved in steroid binding, enzyme binding, zinc ion binding, bile acid binding, carbonate dehydratase activity, identical protein binding, phosphotyrosine binding, transcription factor binding, indanol dehydrogenase activity, PTB domain binding, NF-kappaB binding, cyclin binding, histone kinase activity, and estrogen receptor activity (Figure 2C). KEGG pathway analysis revealed that key targets were involved in cancer-related pathways, chemical carcinogenesis, EGFR tyrosine kinase inhibitor resistance, insulin resistance, the relaxin signaling pathway, the HIF-1 signaling pathway, the pI3K-Akt signaling pathway, the FoxO signaling pathway, the ErbB signaling pathway, the estrogen signaling pathway, acute myeloid leukemia, breast cancer, the Rap1 signaling pathway, transcriptional misregulation in cancer, the tumor necrosis factor (TNF) signaling pathway, and Th17 cell differentiation (Figure 2D). The bubble plot of the top 20 significant terms and pathways is displayed in Figure 2.

**FIGURE 3:**
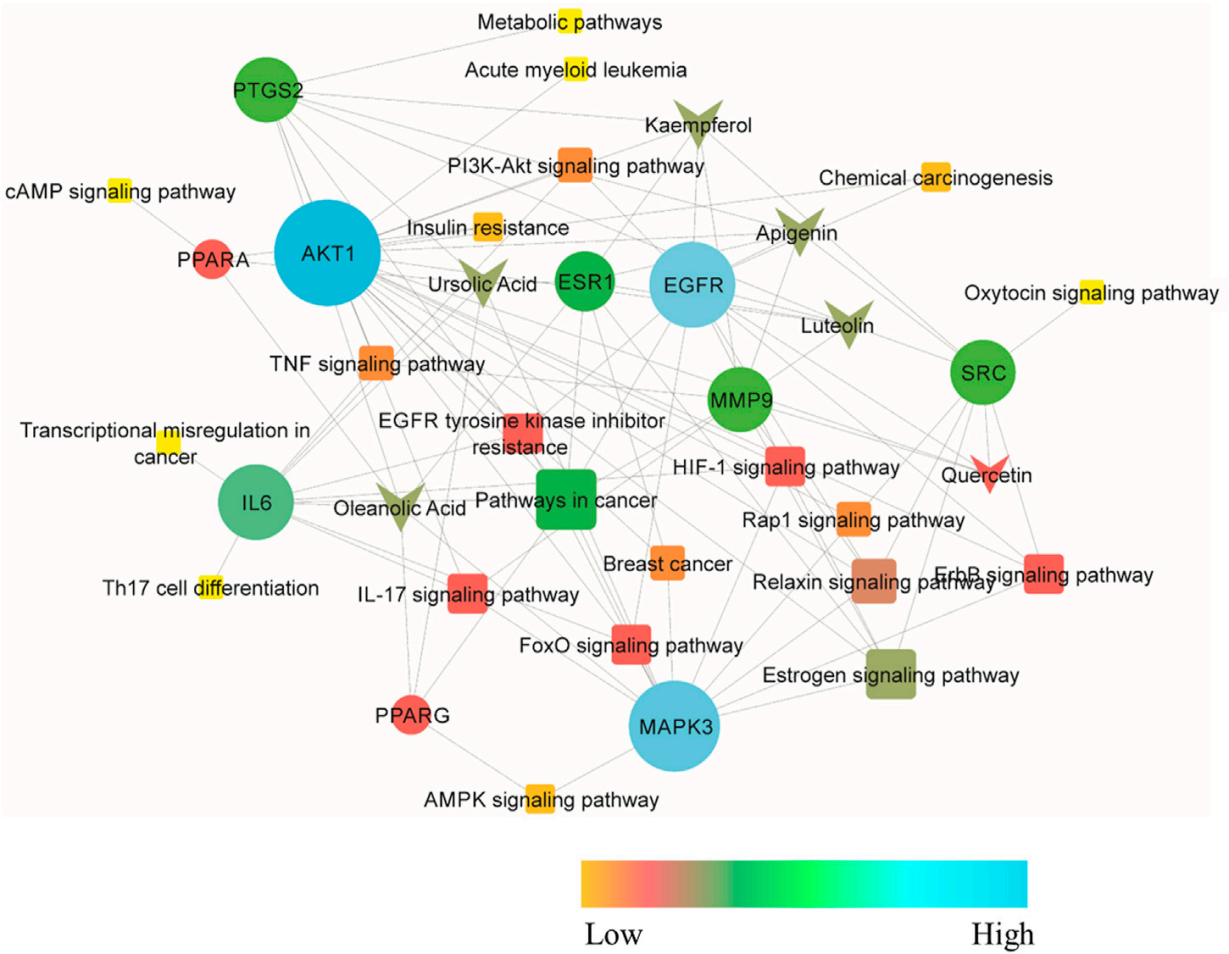
Compound–target–pathway network. The core protein is shown as a circle, active compounds as arrows, and pathways in which the core targets were involved as squares. Additionally, the nodes are colored with a gradient to indicate their degree of connectivity.

The compound–target–pathway network was then constructed to obtain insight into how signaling pathways could be related to the beneficial effect of *C. monogyna* against breast cancer (Figure 3). The compound–target–pathway mainly consists of bioactive compounds, target genes, and their associated pathways. The ultimate goal of presenting the compound–target–pathway network was to better understand the interaction between compounds and disease. Therefore, network analysis indicated that *C. monogyna* could produce anti-breast cancer action by influencing multiple targets and their associated pathways, thus indicating a multi-target pharmacological mechanism of *C. monogyna*-related compounds.

### 3.4 Microarray data analysis

**FIGURE 4:**
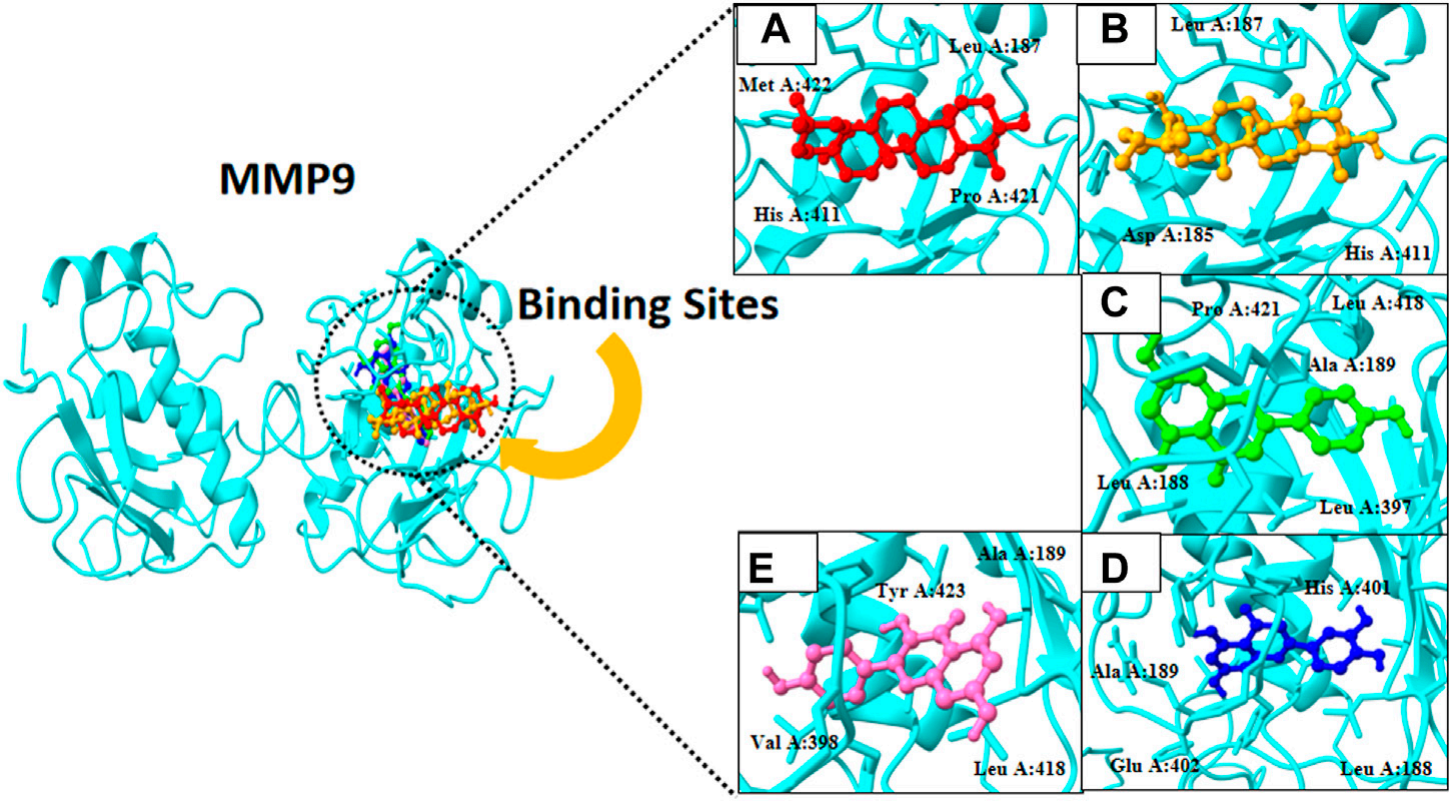
3D visualization of MMP9 protein active compounds: **(A)** Oleanolic acid, **(B)** ursolic acid, **(C)** apigenin, **(D)** oleanolic acid, and **(E)** kaempferol.

The gene expression level of hub genes was then analyzed using the GSE36295 dataset. The GSE36295 dataset contains data from five healthy and 45 breast cancer-affected individuals. The DEGs were then identified using the Limma package of R. Only those genes were considered as DEGs that met the criteria of adj. *p*-value < 0.05 and |log(FC)| ≥ 1, |log(FC)| < −1.0 (Figure 4). The Limma package yielded 1419 DEGs (789 upregulated and 620 downregulated genes). The core targets were then compared with the DEGs, and only two targets, PPARG and MMP9, were found to be overlapped. Furthermore, the expression level of PPARG was upregulated, while the expression level of MMP9 was downregulated.

**FIGURE 5:**
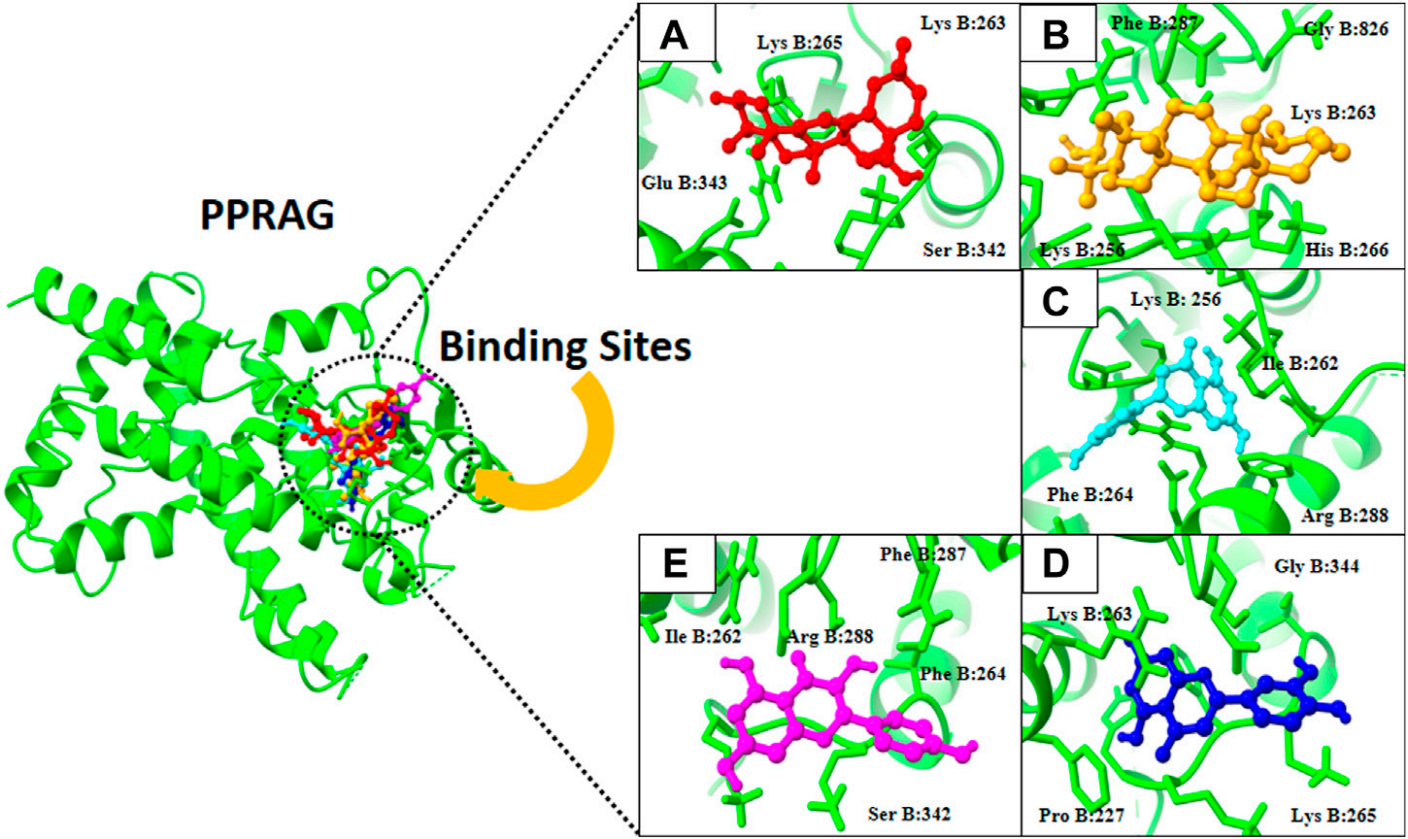
3D visualization of PPRAG protein with active compounds: **(A)** Kaempferol, **(B)** ursolic acid, **(C)** apigenin, **(D)** oleanolic acid, and **(E)** luteolin.

Finally, docking analysis was performed to analyze interactions between core targets and active compounds. The 3D structures of MMP9 and PPARG were downloaded from PDB using pdb id: 1gkc and 1nyx, respectively. The CASTp tool was used to predict the binding pockets of target proteins. The CASTp score used for MMP9 and PPARG were area (SA): 472.041 Å^2^; volume (SA): 274.241 Å^3^, and area (SA): 414.294 Å^2^; volume (SA): 427.042 Å^3^, respectively. Later, the target docking was performed through PyRx. In PyRx, the docking grids of MMP9 and PPRAG were X: −5.5563, Y: −12.8049, Z: −23.3022 and X: 20.9288, Y: 18.4992, Z: 30.0441, respectively, from the center with exhaustiveness equal to 8. The findings of docking analysis revealed that strong binding affinity exists between core targets and active compounds (Table 4). MMP9 has a maximum binding affinity with oleanolic acid (−17.69 kcal/mol) and apigenin (−15.09 kcal/mol). In contrast, PPARG has the highest binding affinity with kaempferol (−16.72 kcal/mol) and ursolic acid (−16.11 kcal/mol). These results indicate that core targets bind stably with active compounds and can be used as a promising candidate for treatment of breast cancer. 3D visualization of the active compounds and target genes is shown in Figure 4 and Figure 5.

**TABLE 4:** Binding affinity and RMSD values of core targets with active compounds.

Compound IDs	Compound name	Binding affinity (kcal/mol)	RMSD	Interacting residues
MMP9 (pdb id: 1gkc)				
10494	Oleanolic acid	−17.69 (kcal/mol)	1.01	His A:411, Pro A:421, Met A:422, and Leu A:187
64945	Ursolic acid	−15.03 (kcal/mol)	2.81	Asp A:185, His A:411, and Leu A:187
5280443	Apigenin	−15.09 (kcal/mol)	1.06	Ala A:189, Pro A:421, Leu A:188, Leu A:397, and Leu A:418
5280445	Luteolin	−14.63 (kcal/mol)	1.55	Ala A:189, Glu A:402, His A:401, and Leu A:188
5280863	Kaempferol	−12.39 (kcal/mol)	1.62	Tyr A:423, Val A:398, Leu A:418, and Ala A:189
PPARG (Pdb id: 1nyx)				
5280863	Kaempferol	−16.72 (kcal/mol)	0.97	Lys B:265, Glu B:343, Lys B:263, and Ser B:342
64945	Ursolic acid	−16.11 (kcal/mol)	1.45	Lys B:263, Lys B:256, Phe B:287, His B:266, and Gly B:826
5280443	Apigenin	−14.49 (kcal/mol)	2.03	Ile B:262, Phe B:264, Arg B:288, and Lys B: 256
10494	Oleanolic acid	−12.17 (kcal/mol)	2.06	Pro B:227, Lys B:263, Lys B:265, and Gly B:344
5280445	Luteolin	−11.95 (kcal/mol)	1.23	Ile B:262, Ser B:342, Arg B:288, Phe B:264, and Phe B:287

### 3.5 Molecular dynamic simulation

**FIGURE 6:**
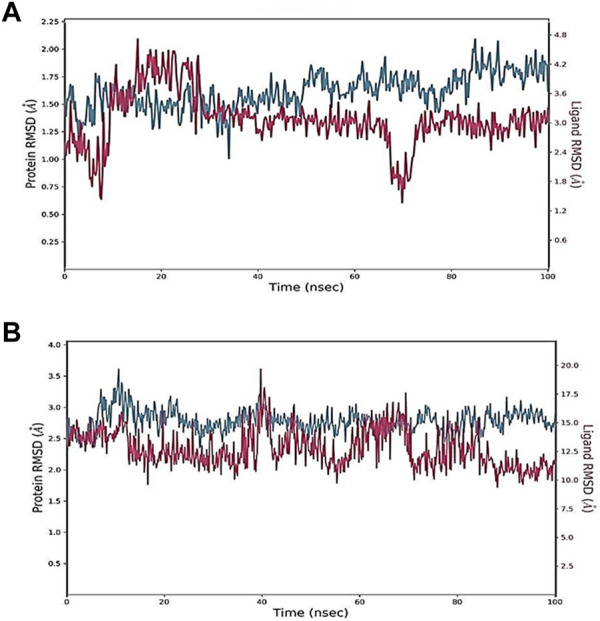
Root mean square deviation (RMSD) of docked complexes: **(A)** Oleanolic acid-MMP9 and **(B)** kaempferol-PPARG.

MD simulations have been conducted in many previously published studies to investigate the validity of docked complexes (Mahmud et al., 2021; Hasan et al., 2022). MD simulation is a strong biophysical technique that can uncover proper binding conformations and other vital dynamic values of ligand–protein interactions. Therefore, it is an important technique in computer-aided drug design (Liu et al., 2019; Michael et al., 2021). MD simulations have been very successful in recent years in optimizing the docked hits (Vanmeert et al., 2019; Anwar et al., 2020; Guterres and Im, 2020). The extensive data generated by MD simulation provides a valuable resource for drug design, offering a comprehensive understanding of the complex interactions between ligands and proteins. In the current study, the selected compounds were considered for 100 ns of MD simulations, and various trajectories were obtained to analyze the results, as recommended. First, the RMSD values of complexes were determined to estimate the structural distance between different coordinates; that is, the RMSD represents the mean distance between atoms at a particular site on the protein. In other words, RMSD values code for assessing the structural integrity of a protein–ligand docked complex. Overall, in the current experiment, the RMSDs of all studied complexes were reported to be stable. The RMSD value increased from 0 to 100 ns with small fluctuations in all of the studied complexes. The average RMSD value of oleanolic acid with MMP9 was found to be 1.5 Å from 30 to 50 ns, which reduced to 0.3 to 0.4 Å at 50–70 ns (Figure 6). In the docked kaempferol and PPARG complex, a variance of 0.5 Å was found at 05–40 ns. At 100 ns, the kaempferol and PPARG complex showed a variance of 0.3 Å. Briefly, the RMSD value of all studied complexes was within the acceptable range.

**FIGURE 7:**
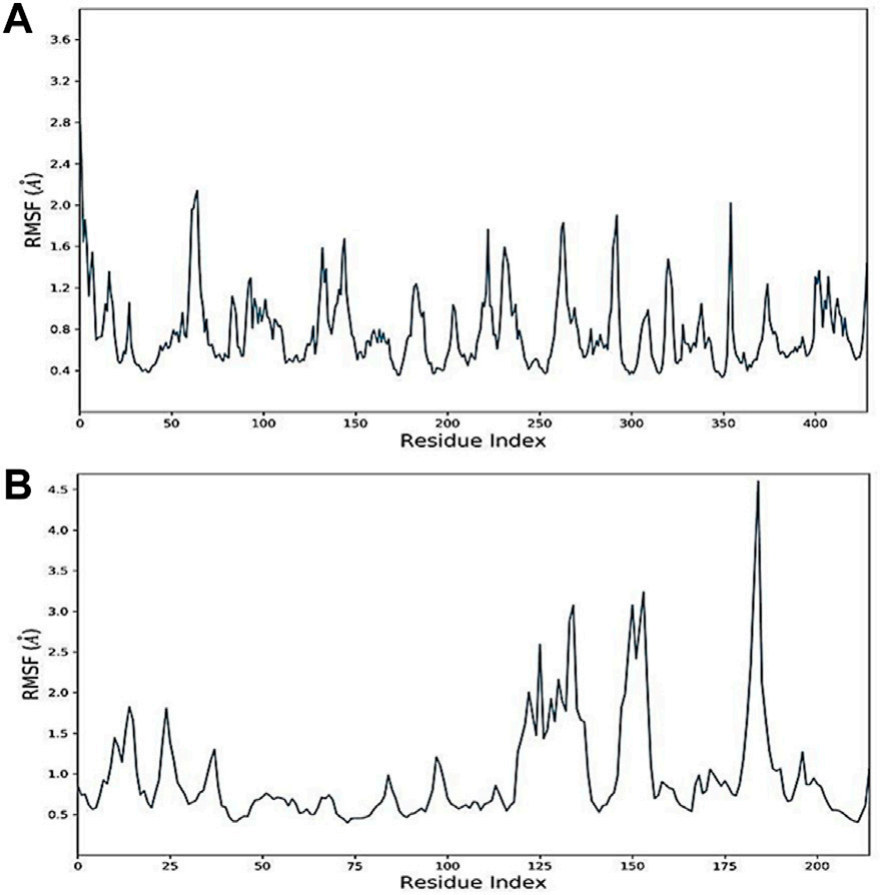
Root mean square fluctuation (RMSF) of docked complexes: **(A)** Oleanolic acid-MMP9 and **(B)** kaempferol-PPARG.

The RMSF values of protein–ligand complexes were generated to further assess residual protein-level flexibility. RMSF predicts the mean fluctuation of a particular amino acid residue from its time-averaged position over time. Docked-complex RMSF plots are demonstrated in Figure 7. The docked complexes of oleanolic acid with MMP9 and PPARG with kaempferol showed RMSF values ranging from 0.4 ± 0.8 Å and 0.9 ± 1.5 Å, which demonstrate the high level of stability between the molecules of proteins in complex with ligands.

**FIGURE 8:**
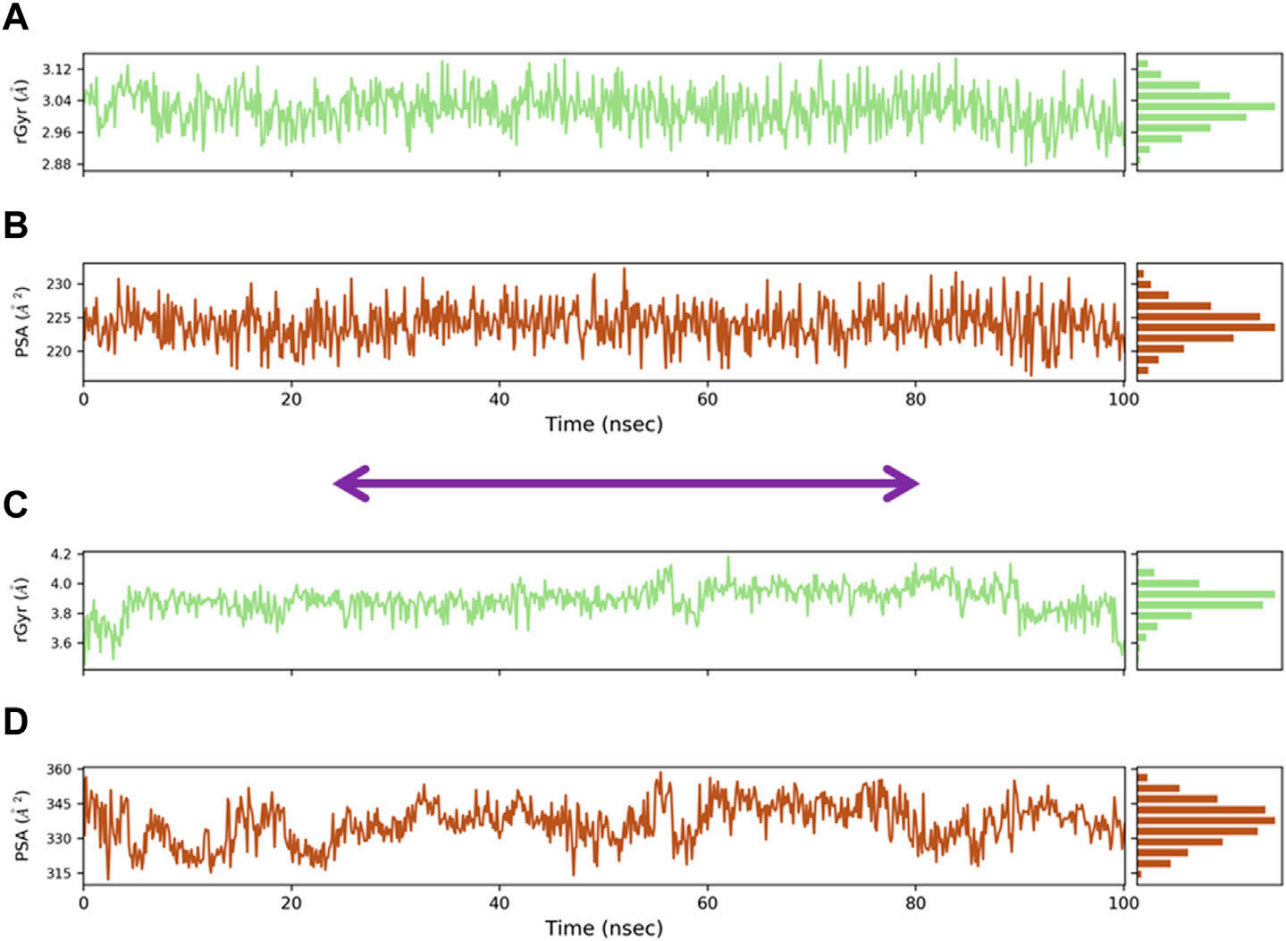
Radius of gyration (Rg) of docked complexes: **(A)** Radius of gyration of oleanolic acid complex, **(B)** PSA for oleanolic acid complex, **(C)** radius of gyration of kaempferol complex, and **(D)** PSA for kaempferol complex.

The compactness of the protein is described by the radius of gyration (Rg), which was calculated for the protein backbone. Rg is considered a novel method for understanding the shape and stability of compounds and their protein during simulation. Rg values of both oleanolic acid/MMP9 and kaempferol/PPARG complexes are displayed in Figures 8A, C. The average Rg values were as follows: for the oleanolic acid and MMP9 complex, the Rg value was 2.96 ± 3.12 Å, while for the PPARG and kaempferol complex, the Rg value was 3.6 ± 4.0 Å. Overall, all of the protein–compound complexes demonstrated significant compactness over the entire 500 ns of simulations. The area of macromolecules that is accessible to water was determined using the PSA. Calculating the PSA value is crucial in determining conformational changes brought on by complicated interactions. The PSA values of oleanolic acid with MMP9 and PPARG with kaempferol complex were found to be 0.6 ± 1.5 nm^2^ and 0.2 ± 0.8 nm^2^ at 100 ns (Figures 8B, D).

## 4 Discussion

Breast cancer is frequently diagnosed in women, and its mortality rates and incidence are expected to increase significantly in the coming years (Momenimovahed and Salehiniya, 2019; Lei et al., 2021). According to the latest statistics from the World Health Organization (WHO), breast cancer is responsible for around 15% of all cancer deaths among women worldwide, which is approximately 685,000 deaths per year (Rahman et al., 2019; Prusty et al., 2020). In the United States, breast cancer is considered the second-leading health concern among women, increasing drastically with 39,620 deaths and 232,240 newly diagnosed cases annually (Noor et al., 2021). Breast cancer in males is a relatively rare disease with an incidence rate of < 1% of all breast cancer occurrences (Leon-Ferre et al., 2018; Gucalp et al., 2019). The higher mortality risk in females is due to a significantly greater proportion of stromal and epithelial tissues and less fatty adipose tissue (Nazari and Mukherjee, 2018; Hieken et al., 2022). Breast cancer comprises a heterogeneous group of neoplasms with distinct probabilities of relapse, molecular phenotypes, morphologies, responses to therapy, and overall survival (Kalinowski, 2019; Rakha and Pareja, 2021). The aggressive behavior of breast cancer, limited prognostic and diagnostic methods, multifactorial occurrence, and the high rate of metastasis hinder the development of effective treatment options for breast cancer (Francies et al., 2020).

The current therapeutic strategies for breast cancer depend on several factors, including the subtype of breast cancer, stage of the disease, and patient’s health status. The main treatment modalities for breast cancer include surgery, radiation therapy, chemotherapy, hormonal therapy, and targeted therapy. Although these treatments can be effective in treating breast cancer, they also have adverse side effects. Network-pharmacology-based studies have shown great promise in identifying new therapeutic targets and developing more effective treatment strategies for breast cancer. By constructing and analyzing molecular networks that underlie breast cancer, researchers can identify critical signaling pathways and potential drug targets that are involved in the development and progression of the disease. Recently, Zhao et al. (2021) used network-pharmacology-based approaches to identify a potential drug target for triple-negative breast cancer (TNBC), a subtype of breast cancer that is particularly aggressive and difficult to treat. The researchers identified a protein called ITGA5 that plays a critical role in the growth and spread of TNBC cells. They then used a computational approach to screen a large database of existing drugs and identified volasertib, which inhibits ITGA5 and has shown promising results in preclinical studies. In summary, network-pharmacology-based studies have the potential to identify new therapeutic targets and treatment strategies for breast cancer. By analyzing molecular networks that underlie the disease, researchers can identify critical signaling pathways and potential drug targets that can be exploited to develop more effective treatments for breast cancer patients.

*Crataegus monogyna,* also called hawthorn, is a spiny plant of Rosaceae family (Ruiz-Rodríguez et al., 2014). *C. monogyna* is a small tree or semi-evergreen shrub with thorns that grows up to five to 15 m long (Martinelli et al., 2021; Hassan et al., 2022b; Shams ul Hassan et al., 2022). *C. monogyna* are a rich source of natural products that are considered to have anticancer qualities. Shirzadi-Ahodashti et al. (2020) reported that leaf extracts of *C. monogyna* have substantial anticancer properties toward cancer cell lines in humans. Rodrigues et al. (2012) demonstrated that the flower of *C. monogyna* has more bioactivity than the fruit. This flower bioactivity inhibits the growth of cancer cell lines in humans. Flavonoids and phenolic acids are the main active compounds of flower buds of *C. monogyna.* This study identified the anti-breast cancer effect of *C. monogyna* for the development of novel treatment options against breast cancer.

Initially, the data related to *C. monogyna*-related compounds were retrieved from databases and the literature. Later, the compound and disease-related targets were predicted to identify the common targets between *C. monogyna* and breast cancer. KEGG pathway analysis revealed that the key targets were mainly involved in EGFR tyrosine kinase inhibitor resistance, the PPAR signaling pathway, proteoglycans in cancer, the relaxin signaling pathway, the HIF-1 signaling pathway, the FoxO signaling pathway, the ErbB signaling pathway, the estrogen signaling pathway, acute myeloid leukemia, the IL-17 signaling pathway, breast cancer, the AMP-activated protein kinase (AMPK) signaling pathway, the cell cycle, the cAMP signaling pathway, the Ras signaling pathway, and the TNF signaling pathway. Finally, the results were validated using microarray data and docking analysis. Docking analysis demonstrated that MMP9 and PPARG have strong binding affinity with luteolin, apigenin, quercetin, kaempferol, ursolic acid, and oleanolic acid. MD simulation, performed to determine the efficacy of compounds, revealed that selected compounds remained stable throughout 100 ns of simulations.

A pro-inflammatory cytokine known as TNF is more commonly expressed in several types of malignancies. Disturbance in the TNF signaling pathway leads to breast cancer. This cytokine is specifically associated with elevated tumor cell proliferation, a higher degree of malignancy, a greater likelihood of metastasis, and typically, a poor prognosis for patients with breast cancer (Mercogliano et al., 2020). Therefore, targeting the genes involved in the TNF signaling pathway can help block the pathogenesis of breast cancer. Furthermore, AMPK negatively regulates the mTOR signal pathway, suppressing tumor growth and proliferation (Li et al., 2015; ul Hassan et al., 2022). Thus, targeting AMPK signaling can help in breast cancer prevention and treatment. Regarding core targets, it is important to note that MMP9 contributes to the breakdown of the extracellular matrix in various physiological and pathological contexts, including cancer. Huang et al. (Huang, 2018) reported that overexpression of the MMP9 protein is highly associated with breast cancer. Therefore, MMP9 can be used as a biomarker in breast cancer and, ultimately, development of novel treatment options.

To sum up, our study provides a scientific foundation to reveal the *C. monogyna*-related compounds as a promising treatment option for breast cancer. Integration of network pharmacology with bioinformatics analysis revealed multitarget pharmacological mechanism of *C. monogyna* in breast cancer. We validated the results using gene expression data and molecular docking analysis. Further *in vivo* and *in vitro* studies are required to validate the efficacy of current findings. There are several limitations in our study. First, additional tests are required to confirm our findings. Second, a larger database of traditional medicines target genes is required, which would improve the accuracy of the network pharmacology analysis results. Third, even after combining the outcomes of network pharmacology and molecular docking, we could not fully comprehend the precise therapeutic mechanism of *C. monogyna*. A way to navigate around this problem is to develop new bioinformatics tools with novel strategies for ensuring the potential targets and, ultimately, the effectiveness of multitarget drugs. Recently, Yang et al. (Guterres and Im, 2020) proposed a novel feature selection strategy to improve the stability and reproducibility of the discovery of schizophrenia (SCZ) gene signatures. The new strategy was able to identify differentially expressed genes and demonstrated superior stability and differentiating ability compared to previous methods. Fu et al. (2022) and Yang et al. (2020) used NOREVA, which can evaluate processing performance, optimize data processing, and process time-course and multiclass metabolomics data. These tools and methods will be required to further explore and validate the NP approaches. Despite the fact that we have presented some interesting data, additional studies and clinical trials are needed to explore the potential of *C. monogyna* to validate their medicinal usages.

## 5 Conclusion

Breast cancer is a commonly diagnosed cancer and an ongoing challenge as it is liable for the annual death of 685,000 women globally. Due to the complexity of disease heterogeneity, resistance to anticancer drugs, and the need for effective therapeutic targets, researchers and clinicians are seeking promising treatment options to advance precision medicine. Pharmacotherapy that utilizes natural scaffolds is considered a promising and innovative approach. A network pharmacology approach was employed in the current study in conjunction with bioinformatics analysis to investigate the multitarget pharmacological mechanism of *Crataegus monogyna* for breast cancer treatment. Our findings uncovered that active compounds of *C. monogyna*, including luteolin, apigenin, quercetin, kaempferol, ursolic acid, and oleanolic acid, have strong binding affinity with MMP9 and PPARG. Microarray data, docking, and simulation studies strengthened our findings. These targets needs to be further validated using *in vitro* and *in vivo* experiments. These proteins could serve as potential drug targets for the development of new therapeutics that can better target and treat breast cancer. The predicted compounds show multicomponent, multitarget, and multipathway properties. These findings will serve as a baseline for future research into the mechanism of *C. monogyna* against breast cancer.

## Data Availability

The datasets presented in this study can be found in online repositories. The names of the repository/repositories and accession number(s) can be found in the article/Supplementary Material.

## Supplementary material

SUPPLEMENTARY TABLE S1 2D interactions of the target protein MMP9
