# Supplementary material for: Therapeutic effects of Crataegus monogyna inhibitors against breast cancer
Source: Front Pharmacol. 2023 Apr 27;14:1187079. doi: 10.3389/fphar.2023.1187079 (PMC10174464; doi:10.3389/fphar.2023.1187079)
Supplement: Supplementary file 1 [file Table1.docx]

**Supplementary file**

**Table S1:** 2D Interactions of the target protein MMP9

| 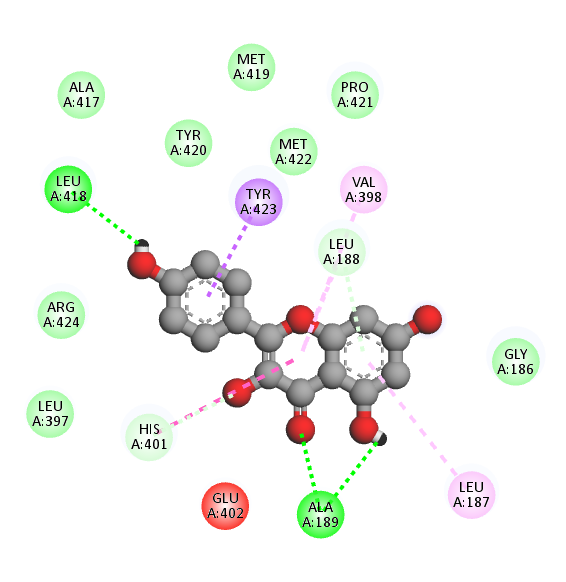  **5280863** |
| --- |
| 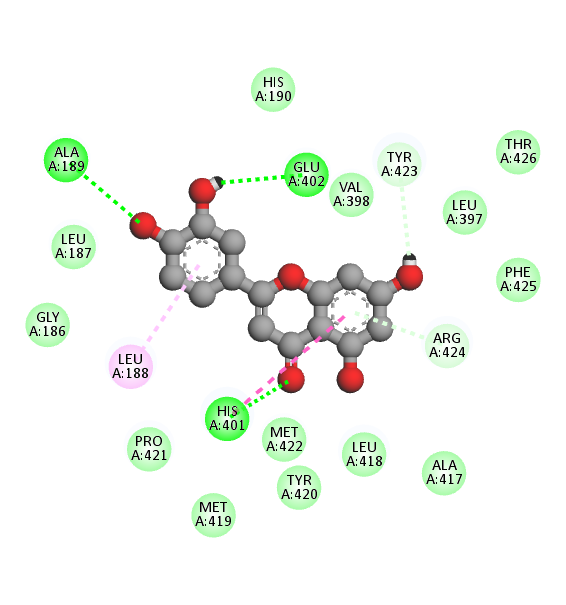  **5280445** |
| 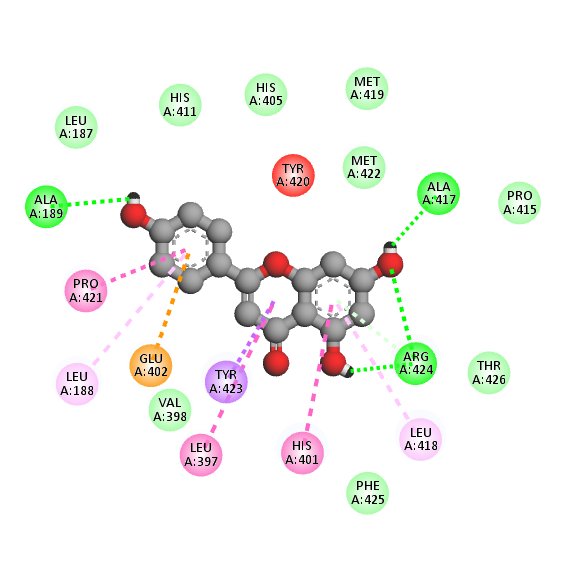  **5280443** |
| 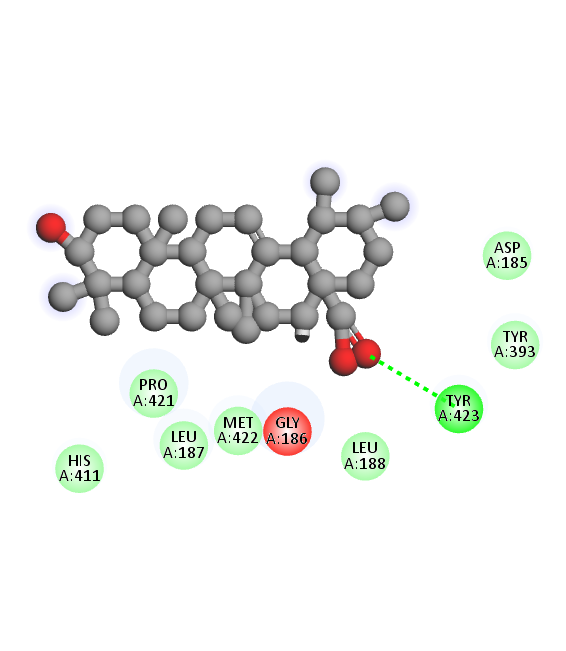  **64945** |
| 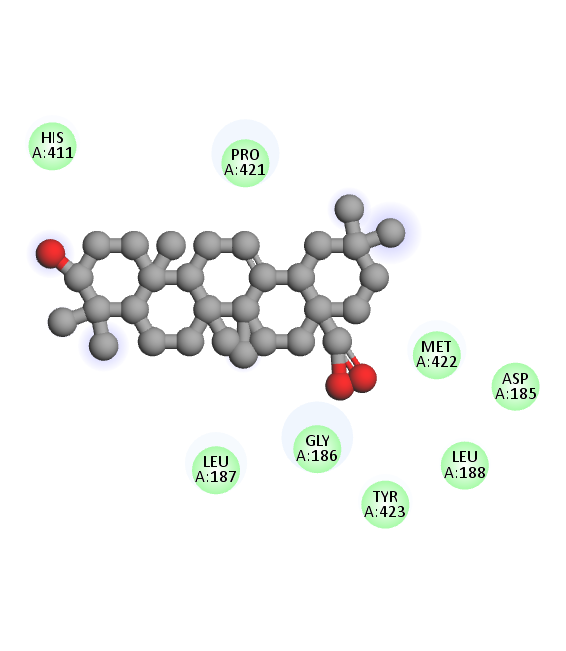  **10494** |

**Table S2:** 2D Interactions of the target protein MMP9

| **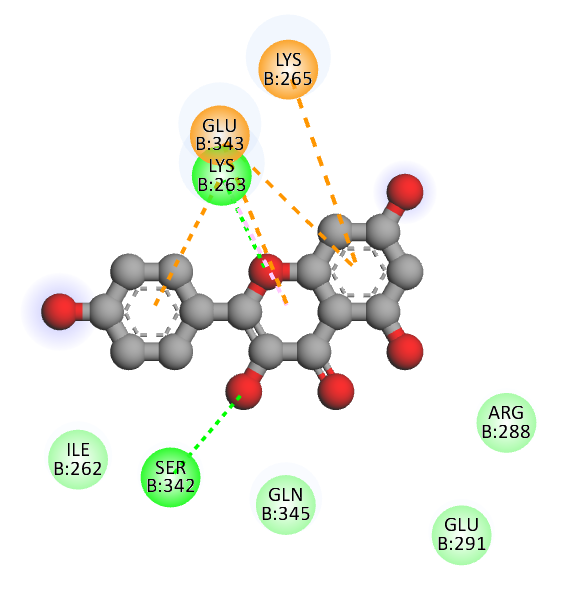**  **5080863** |
| --- |
| 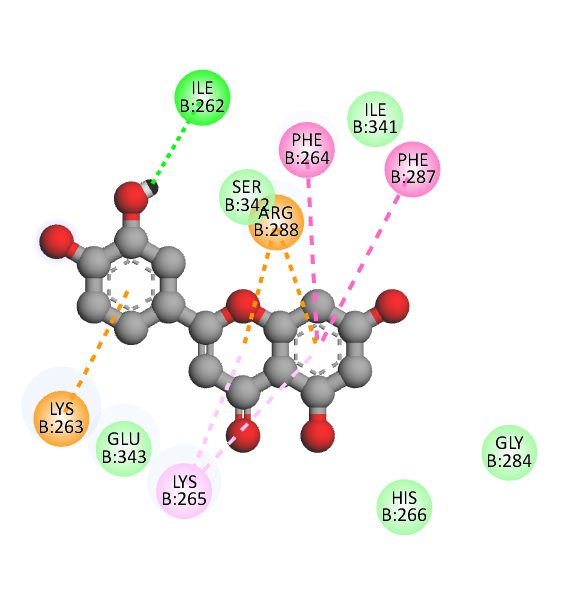  **5280445** |
| 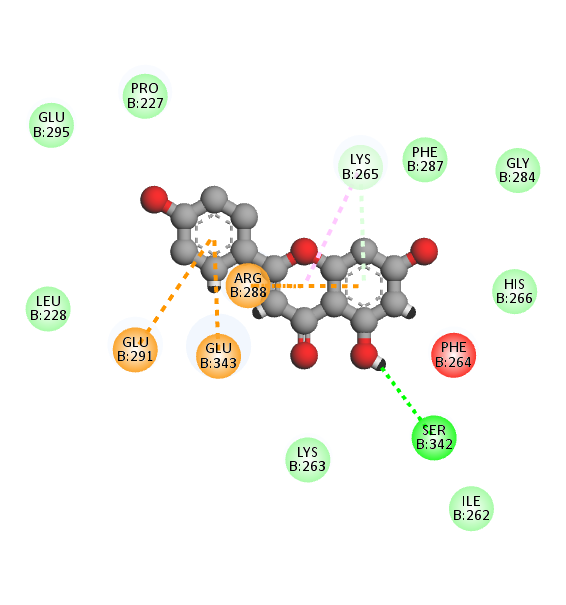  **5280443** |
| 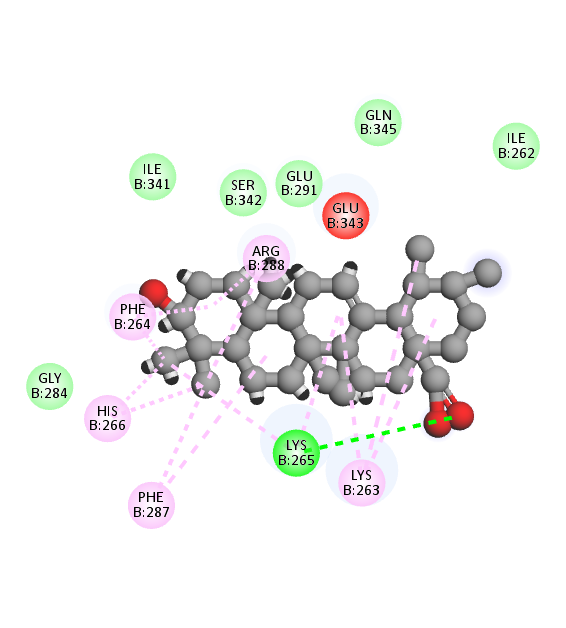  **64945** |
| 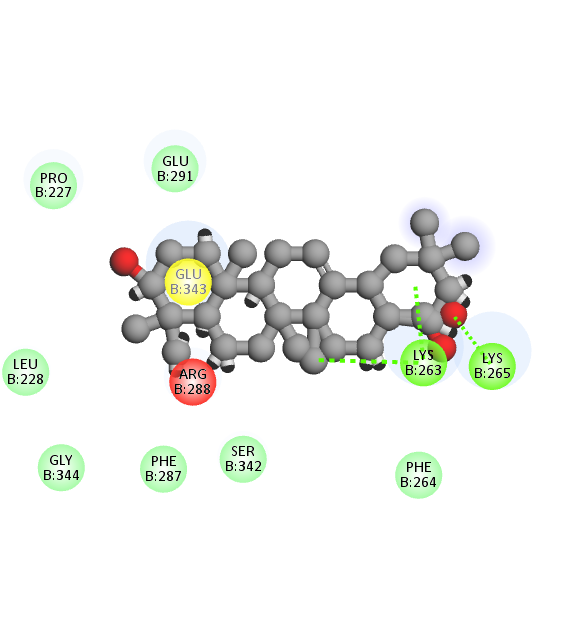  **10494** |
